# Awareness of the impact of sex and gender in the disease risk and outcomes in hematology and medical oncology—a survey of Swiss clinicians

**DOI:** 10.1002/cnr2.1961

**Published:** 2024-01-23

**Authors:** Xenia Darphin, Jeanne Moor, Cristina Espinosa da Silva, Anke Richters, Berna C. Özdemir

**Affiliations:** ^1^ Department of Hematology Spital Limmattal Schlieren Switzerland; ^2^ Department of Internal Medicine Bern University Hospital Bern Switzerland; ^3^ Department of Medicine University of California San Francisco California USA; ^4^ Department of Research and Development The Netherlands Comprehensive Cancer Organisation Utrecht The Netherlands; ^5^ Department of Medical Oncology Bern University Hospital Bern Switzerland

**Keywords:** cancer, gender, hematology, oncology, sex

## Abstract

**Background:**

Although male and female cancer patients are distinct in many ways, there is a limited understanding in the differences between male and female biology and differing pharmacokinetic responses to cancer drugs. In fact, sex and gender are currently not considered in most treatment decisions in the fields of oncology and hematology. The lack of knowledge about potential sex differences in both disciplines may lead to differences in treatment efficacy, toxicity, and the overall survival (OS) of patients.

**Aim:**

To evaluate their awareness about sex and gender in clinical practice we surveyed Swiss hematologists and oncologists from September to November 2022.

**Methods:**

We collected data about the clinical knowledge, experimental research, palliative care, quality of life, as well as the participant perception of the importance of sex and gender. We identified 767 eligible clinicians, of whom 150 completed the survey (20% response rate).

**Results:**

While most participants agreed that sex and gender were relevant when treating patients, it became clear that fewer participants knew about sex and gender differences in treatment toxicity and survival, which in turn would affect the treatment of their patients. Most participants agreed that this topic should be integrated into continuing education and research.

**Conclusion:**

Our findings indicate the need for more awareness and training on sex and gender in cancer research and clinical care among oncologists and hematologists. Ideally, by better educating medical students and health professionals, a demand is created for improving research policies, publications and therefore patient care.

## 1 INTRODUCTION

Individuals differ on many levels, including socially and biologically. Despite the distinction between sex and gender, these terms are often used interchangeably in research and are not sufficiently considered in modern medical practice. Sex refers to biological features such as chromosomes, physiological processes, and organs (including and beyond reproductive ability).^1^ Gender, on the other hand, describes the characteristics of our socially constructed roles, behaviors, and identity.^2^ Most treatment decisions are based on decades‐long research using predominately male cells, animals, and individuals,^3^ which is problematic given the growing body of data indicating sex differences in various diseases (especially pertaining to prevalence, symptoms, diagnosis, and prognosis).^4^

Oncology and hematology are disciplines that currently incorporate the complexities of carcinogenesis and molecular genetic differences in tumor biology in preclinical and clinical research.^5^ Until recently, both fields were tumor type‐centered and aimed to identify common characteristics among patients to determine the best treatment protocol for each patient. The development of precision medicine and the introduction of novel diagnostic tools have allowed a better understanding of genomic subgroups, immunological interactions, and biomarkers in different tumor types.^5^, ^6^ These critical clinical advances have replaced the “one size fits all” tumor type‐centered treatments and general‐purpose cytotoxic drugs with targeted approaches and biomarker‐driven treatments (e.g., tyrosine kinase inhibitors, immunotherapies, adaptive cell therapy, and personalized vaccines).^6^ Moreover, factors like age, frailty, organ function, and concomitant drugs are often considered to further personalize treatment decisions.

Despite these advances, there is limited understanding regarding the differences between male and female biology and differing pharmacokinetic responses to cancer drugs. Most knowledge about tumor biology and anticancer drugs is still based on the male physiology in cells, animals, and humans. Historically, females have been underrepresented and underreported in biomedical research and clinical trials^7^ due to the potential effect of cyclic hormonal changes on results, fertility risk, and additional pregnancy‐related considerations. Yet, there are notable and significant sex differences that should be considered when tailoring anticancer therapies. As an example, a male body mass is comprised of 80% of lean, metabolically active muscle while that constitutes only 65% of female body mass.^8^ The higher fraction of adipose tissue in the female body may lead to higher rates of toxicity, which may require dose reductions during treatment and could also lead to worse health outcomes compared to male patients. In spite of the notable physiological differences between the sexes, in current clinical practice male and female patients receive the same anticancer treatment regimens and medication dosages.

In clinical studies, there is a distinct lack of reporting about sex differences pertaining to tumor biology, mutational markers, as well as treatment response and adverse effects.^8^, ^9^ Addressing this significant knowledge gap in both disciplines could improve sex‐specific dosing, treatment efficacy, toxicity, and the OS in both sexes. To address this knowledge gap and raise awareness regarding the need for reporting the sex and gender differences in non‐sex‐related malignancies, we developed a survey for Swiss oncologists and hematologists to assess their current knowledge about the impact of sex and gender in disease risk and outcomes, specifically in clinical practice. In addition to raising awareness about this issue, we aim to motivate clinicians and researchers to be more critical about sex and gender differences in education and daily practice, and to also consider policy changes in basic research, clinical trial conduct and reporting.

## 2 METHODS

Our cross‐sectional online study was conducted among hematologists and oncologists in Switzerland. To identify potential participants, we used web searches to generate a list of clinicians in hematological and oncological departments in hospitals and medical practices in Switzerland, which identified 56 institutions within Switzerland and 767 eligible clinicians (245 hematologists and 522 oncologists). To recruit potential participants, we emailed 767 identified clinicians in September 2022 with a description of our cross‐sectional study and a link to participate via SurveyMonkey® (an online platform). Two weeks after this initial email, we sent a follow‐up email to all individuals in the email listing, except to those who already participated or those who opted out of the email listing. In November 2022, we also handed out a flyer at the Swiss Oncologists and Hematologist Congress (SOHC) with information about the study and a QR code to participate in the survey. Our online survey was available over the course of 10 weeks (from September 19 to November 26, 2022), and was closed at the end of the study period.

### 2.1 Survey instrument

The survey collected data including participant demographics and career‐related questions such as working region within Switzerland and clinical fields of work. Using published literature in the fields of hematology and oncology regarding established sex and gender differences, we also developed questions related to to clinical knowledge in the areas of hematology, oncology, experimental research, palliative care, quality of life in older populations, and participant perceptions regarding the general importance of sex and gender in cancer‐related treatment options. As these questions were developed specifically for our study, we pilot‐tested them in a small group of clinicians from other medical fields to determine whether participants could understand the questions and to avoid creating a knowledge bias in our target study population. We initially developed the survey questions in English. Two translators then translated them into German and French using forward translation. We then back‐translated the survey questions to English using a free online translating tool (DeepL®) to assess the accuracy of the German and French translations.

For the questions developed to assess participant awareness of sex and gender in disease risk and outcomes, we used five‐point Likert‐type responses to measure the participant's level of agreement. After a participant answered a specific question, the online survey would then provide the correct response with the corresponding literature citation.

### 2.2 Statistical analyses

Given our study objectives and small sample size, our analysis focused on descriptive statistics. We used the medians and interquartile ranges to describe the distribution of skewed continuous variables, and reported proportions for categorical variables. We reported descriptive statistics for the sample overall and sex‐stratified.

## 3 RESULTS

A total of 150 clinicians completed the survey, which corresponded to a 20% response rate. Most participants (82%) worked in the German‐speaking region of Switzerland, and 99% worked in a hospital setting (Table 1). Approximately half of the participants were biologically female (53%), and 76% were aged between 31 and 50 years. All of the participants in our sample reported concordance with their sex assigned at birth and their gender expression. To be consistent, we will refer to the participants from this study as female or male. While most participants indicated knowing the difference between the sex and gender, biological sex was considered twice as relevant as sociocultural gender role and responses seemed comparable between male and female participants.

**TABLE 1:** Characteristics of survey participants, *n* (%).

Characteristics			Sex			
	Total (*N* = 150)		Female (*n* = 80)		Male (*n* = 70)	
Age						
Median (IQR)	41	35–48	39	34–44	45	39–53
≤30	6	4%	6	8%	0	0%
31–40	67	45%	43	54%	24	34%
41–50	46	31%	23	29%	23	33%
51–60	24	16%	7	9%	17	24%
≥61	7	4%	1	0%	6	9%
Gender expression						
Very feminine	13	9%	13	16%	—	—
Feminine	65	43%	65	81%	—	—
Between feminine and androgynous/neutral	2	1%	2	3%	—	—
Between masculine and androgynous/neutral	5	3%	—	—	5	7%
Masculine	56	37%	—	—	56	80%
Very masculine	9	6%	—	—	9	13%
Country and the region of work						
Switzerland, French‐speaking region	23	15%	11	14%	12	17%
Switzerland, German‐speaking region	123	82%	67	84%	56	80%
Switzerland, Italian‐speaking region	4	3%	2	3%	2	3%
Work setting^a^						
Group practice	7	5%	3	4%	4	6%
Hospital	76	51%	42	53%	34	49%
University hospital	74	49%	38	48%	36	51%
Research	13	9%	5	6%	8	11%
Other	2	1%	—	—	2	3%
Date of medical licensing exam						
1980–1989	13	9%	2	2%	11	16%
1990–1999	22	15%	8	10%	14	20%
2000–2009	53	35%	30	38%	23	33%
2010–2019	62	41%	40	50%	22	31%
Job position						
Resident physician	33	22%	24	30%	9	13%
Attending physician/consultant	53	35%	36	45%	17	24%
Chief physician/head of the department	60	40%	18	23%	42	60%
Other	4	3%	2	3%	2	3%
Clinical field						
Oncology	73	49%	36	45%	37	53%
Hematology	62	41%	35	44%	27	39%
Both oncology and hematology	15	10%	9	11%	6	9%

^a^ Categories are not mutually exclusive.

In some of our sex‐stratified analyses, we found that resident or attending physicians comprised 75% of female and 37% of male participants, respectively (Table 1). Among the male participants, 60% were chief physicians or heads of departments, compared to only 23% among female participants.

**FIGURE 1:**
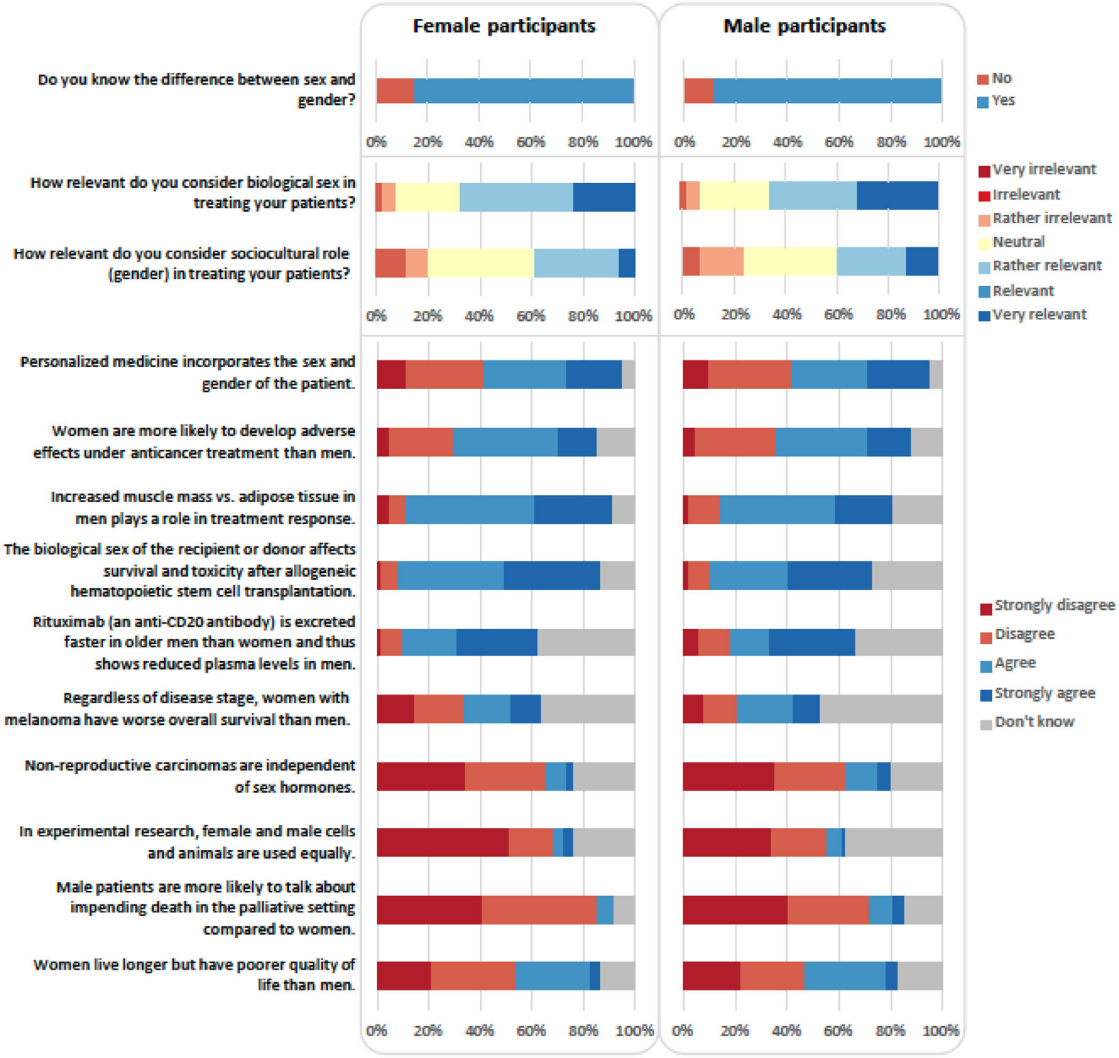
Knowledge and perceived relevance of the sex and gender and agreement to statements on sex and gender in cancer research and clinical practice.

Half (54%) of the participants in our study agreed that sex and gender should be incorporated in personalized medicine to be accurate, although only 23% strongly agreed with this statement (Figure 1; Table 2). Over half (59%) of our sample knew about the predominate use of male cells and animals in experimental research, although one‐third (34% male vs. 32% female) reported that they were unaware of these disparities.

**TABLE 2:** Survey questions regarding sex and gender in cancer research and clinical practice.

Statement	Correct response [Citation]
Do you know the difference between sex and gender?	—
How relevant do you consider biological sex in treating your patients?	—
How relevant do you consider the sociocultural role (gender) in treating your patients?	—
Personalized medicine incorporates the sex and gender of the patient.	FALSE^10^
Women are more likely to develop adverse effects under anticancer treatment than men.	TRUE^8^, ^11^, ^12^
Increased muscle mass vs. adipose tissue in men plays a role in treatment response.	TRUE^13^, ^14^
The biological sex of the recipient or donor affects survival and toxicity after allogeneic hematopoietic stem cell transplantation.	TRUE^15^
Rituximab (an anti‐CD20 antibody), used in the therapy of lymphomas among other malignancies, is excreted faster in older men than women and thus shows reduced plasma levels in men.	TRUE^16^
Regardless of the disease stage, women with melanoma have worse OS than that in men.	FALSE^17^, ^18^, ^19^
Nonreproductive carcinomas are independent of sex hormones.	FALSE^20^, ^21^
In experimental research, female and male cells and animals are used equally.	FALSE^22^, ^23^, ^24^, ^25^
Male patients are more likely to talk about impending death in the palliative setting compared to women.	FALSE^26^, ^27^, ^28^
Women live longer but have poorer quality of life than that in men.	TRUE^29^

In regards to sex disparities in antitumor treatment, 54% agreed that women are more likely to develop adverse effects from anticancer treatments (Figure 1; Table 2). 11% and 15% of male and female participants, respectively, were unaware of the greater burden of adverse events (AEs) among women. Nearly 40% of participants (44% male vs. 35% female) were unaware of sex differences in the OS in melanoma. Most participants (64% male vs. 77% female) agreed that muscle mass and adipose tissue can affect treatment response. Over half of our participants (63% female and 59% male participants) disagreed that nonreproductive carcinomas are independent of sex hormones. However, 15% and 10% of male and female participants, respectively, agreed with this statement.

Approximately one‐third of the sample knew that rituximab (i.e., an anti‐CD20 antibody) showed reduced plasma levels among male compared to female patients (Figure 1; Table 2). However, 17% of our male participants and 10% of our female participants disagreed with this statement. Regarding stem cell transplantations, 75% and 60% of female and male participants, respectively agreed that survival and toxicity is affected by the biological sex of the recipient or donor.

More female participants (82% compared to 66% male participants) agreed that male patients in palliative settings discuss impending death more than female patients (Figure 1; Table 2). Nearly half (48%) of our sample disagreed that older women had poorer quality of life (43% male vs. 51% female), and 14% of participants responded that they were unsure.

At the end of the survey, 42% of participants (37% male vs. 46% female) agreed that the information provided in our survey changed their opinions about the relevance of sex and gender in everyday clinical practice. Furthermore, most participants indicated that they would like to see this topic integrated into continuing education (74%) and research (83%). Among female participants, 85% (compared to 61% of male participants) indicated that they wanted sex and gender integrated into continuing education, and 90% (compared to 69% of male participants) of wanted these topics integrated in research.

## 4 DISCUSSION

The results from our cross‐sectional online survey indicate that there is room for improved awareness and education regarding sex and gender in cancer research and patient care among Swiss hematologists and oncologists. While a notable proportion of clinicians responded incorrectly to certain statements or indicated that they were unsure of the correct response, there seems to be an important opportunity to raise awareness about sex and gender disparities given that nearly half of the sample indicated that the information from our survey changed their opinions about the relevance of sex and gender in daily clinical practice. Most participants were aware of the difference between the two terms and considered sex and gender as part of “personalized medicine.” However, currently personalized or precision medicine aims to identify molecular and biological characteristics in most cases to customize patient‐specific targeted treatments, and sex and gender are not typically considered.^10^

The difficulty with assessing sex and gender begins at the preclinical research level. We recently reported in an international survey among academic cancer researchers that half of the 1247 researchers did not know the sex of the cell lines used in their research, even though data suggest that the sex of cell lines can affect the results of in vitro experiments.^30^, ^31^ This was also reflected in the responses from our current study, given that nearly 40% of male participants did not know about this bias. As a further example, Nunes and colleagues showed that higher levels of toxicity were inflicted upon male‐derived cells in an anticancer high throughput screening, which presented a sex‐related difference in cell sensitivity to 79 out of 81 antineoplastic agents.^32^ Similarly, as nearly two‐thirds of the participants in our study recognized, there is increasing evidence that sex chromosomes and hormones play an important role in development of various nonsex‐dependent cancers (such as melanoma, lung, bladder, and liver cancer).^33^, ^34^

As recognized by over half of our participants, women are more likely to develop AEs from anticancer treatment. Indeed, women often have higher blood drug concentrations and longer elimination times than men with the same drug dose,^35^ leading to a higher risk for adverse drug reactions across all drug classes and higher hospitalization rates among women.^36^, ^37^ For example, The SEXIE‐R‐CHOP‐14 trial showed that elderly men treated for diffuse large B‐cell lymphoma (DLBCL) had lower serum levels of the anti‐CD20‐antibody rituximab. By increasing the usual dosage for elderly men from 350 to 500 mg/m^2^, the progression‐free survival and the OS improved compared to previous trials.^16^ Although this data has not been incorporated into newer trial designs,^38^, ^39^, ^40^ on a positive note, the 2023 National Comprehensive Cancer Network (NCCN) guidelines for the treatment of DLBCL now recommend higher doses in men over 60 years of age receiving the R‐CHOP21 regimen.

Similarly, women have a reduced clearance of 5‐Fluorouracil (5‐FU), a drug commonly used in treating gastrointestinal cancers, leading to higher exposure and subsequently higher toxicity which is mainly hematological.^41^, ^42^ In a systematic review of AEs in clinical trials, Unger et al. reported that women had a 34% increased risk of severe toxicity for all treatment types (cytotoxic drugs, immunotherapies, and targeted therapies).^43^ To prevent infections in cases of neutropenia, hematopoietic growth factors (G‐CSF) can be applied to stimulate the maturation and mobilization of granulocytes in the bone marrow. However, the current NCCN^44^ guidelines for G‐CSF administration do not acknowledge differences between men and women in the prophylactic and therapeutic setting.^44^ Similarly, the Multinational Association of Supportive Care in Cancer (MASCC) febrile neutropenia risk index does not incorporate sex and gender in risk stratification, leading to a lack of inclusion in the European Society for Medical Oncology (ESMO) guidelines for management of febrile neutropenia.^45^ Increased risk for gastrointestinal AEs, such as nausea and vomiting, have also been reported among women receiving anticancer treatments.^12^, ^46^ However, this is neither mentioned in the current American Society for Clinical Oncology (ASCO) Antiemetics Guidelines, nor are there any sex‐specific recommendations for preventing and treating treatment‐related nausea and emesis.^47^ The lack of sex‐adjusted data and guidelines creates a vacuum in clinical practice, which in turn makes anticancer treatment options imprecise in daily practice.

The effects on the immune system and immune responses are becoming more apparent due to the implementation of immune checkpoint inhibitors in anticancer treatment. For instance, in the KEYNOTE‐024 trial, male non small‐celllung cancer (NSCLC) patients derived a significant benefit from the immune checkpoint inhibitor pembrolizumab compared to standard chemotherapy (hazard ratio [HR] for progression or death = 0.39, 95% Confidence Interval [CI]: 0.26–0.58) while this benefit was substantially lower among female patients (HR = 0.75, 95% CI: 0.46–1.21) in the sub‐group analysis.^48^, ^49^ In line with this, lower benefit was reported for women with advanced melanoma receiving combined immune checkpoint inhibitors.^50^ Given that the clinical trials are not designed or powered to investigate potential sex differences in treatment effects, no meaningful conclusions can be drawn. Some meta‐analyses comparing different immune checkpoints in various tumor types have suggested sex differences in the efficacy of these therapies, while others did not find any significant differences.^51^, ^52^, ^53^ Pooled analyses of individual patient data from clinical trials could help to address this question until prospective trials are designed with adequate power to show sex differences.

Our study had several limitations. In our questionnaire, we mainly focused on binary biological sex rather than nonbinary gender given that we developed our survey questions from previously published literature emphasizing biological sex. The lack of information on gender in published studies did not allow for incorporating more gender‐related questions, which is a self‐perpetuating problem and a limitation of our study. While many treatment regimens, past and present, are applied intravenously, the current trend towards orally available anticancer treatments might make behavioral differences a critical consideration. Among patients with cardiovascular diseases, such as hypertension, diabetes, and hyperlipidemia, men have been shown to have higher adherence rates than women.^54^, ^55^ This could also apply to our patient population, which in turn requires us to consider gender differences more in daily clinical practice. Another imitation of our survey was the convenience sampling used to recruit our study sample. Given that many hospitals and medical practices did not include their residents on their web pages, we may have sampled more experienced physicians which might cause a bias in participant knowledge. We certainly observed demographic trends given that over half of our female participants were under the age of 40 years and in the beginning of their careers while over half of the male participants were over 40 years old and had higher positions within the hospital settings. The same difficulty occurred when searching for medical institutions in Switzerland's French and Italian regions, which may have resulted in oversampling of German‐speaking clinicians. Our study is also prone to selection bias, given our low response rate, as well as nonresponse bias. Clinicians interested in sex and gender differences might have been more prone to participating in our survey. Using the survey as an educational instrument and providing the answers might have created a bias when answering the subsequent question.

Taken together, most participants were interested in the topic of sex and gender, had a basic knowledge of theoretical sex differences, but did not have solid information to apply in clinical practice. As the above stated literature and our survey results show, there is an increasing amount of published data concerning the differences between the sexes although it still needs to be implemented in daily clinical practice. More female participants need to be included in research and sex‐adjusted subgroup analysis must be reported. Notably, more education and studies concerning sex‐ and gender differences are necessary in the medical field. We are convinced that increased awareness and training on sex and gender differences in hemato‐oncology are required to ultimately increase consideration of these two critical factors in clinical trial design and treatment decisions and improve the outcome of both male and female patients.

## AUTHOR CONTRIBUTIONS

All authors had full access to the data in the study and take responsibility for the integrity of the data and the accuracy of the data analysis. Conceptualization, Berna C. Özdemir; Methodology Xenia Darphin, Jeanne Moor, Berna C. Özdemir; Investigation, Darphin Formal Analysis Darphin, Anke Richters. Writing–Original Draft, Darphin, CED, Berna C. Özdemir Writing–Review & Editing, Darphin, Jeanne Moor, CED, Anke Richters, Berna C. Özdemir Visualization, Anke Richters Supervision Berna C. Özdemir.

## CONFLICT OF INTEREST STATEMENT

The authors do not have any competing interests to declare in relation to this work.

## ETHICS STATEMENT

A Clarification of Responsibility with the Cantonal Ethics Committee Bern (BASEC Nr. Req‐2022‐00436) indicated that our study did not require approval by an ethics committee. We assumed that participants consented to participate in our study after reading a description of our study and voluntarily clicking on the survey link to participate. This work was not supporting by any funding.
